# Case Report: An Adult Patient With Deficiency of Adenosine Deaminase 2 Resembled Unilateral Frosted Branch Angiitis

**DOI:** 10.3389/fmed.2021.642454

**Published:** 2021-04-29

**Authors:** Yufeng Xu, Yi Shan, Yin Hu, Jing Cao, Yijie Wang, Lixia Lou, Panpan Ye

**Affiliations:** ^1^Eye Center, College of Medicine, The Second Affiliated Hospital of Zhejiang University, Zhejiang, China; ^2^Department of Neurology, College of Medicine, The Second Affiliated Hospital of Zhejiang University, Zhejiang, China

**Keywords:** deficiency of adenosine deaminase 2, frosted branch angiitis, autoinflammatory and autoimmune diseases, retina vascular disorder, blood coagulation factor XII

## Abstract

**Purpose:** Deficiency of adenosine deaminase 2 (DADA2) is a rare autosomal recessive systemic autoinflammatory disorder. We describe a rare case of an adult patient with DADA2 who presented with unilateral frosted branch angiitis (FBA) combined with branch retinal vein occlusion and panuveitis.

**Method:** This paper is a clinical case report.

**Results:** A 31-year-old male patient complained of blurred vision in his right eye for 2 days. His fundus examination showed FBA combined with branch retinal vein occlusion and panuveitis. He had a medical history of intermittent and recurrent fever, skin rash and aphthous ulcer for 5 years, and lacunar infarction for 1 month. Laboratory examinations showed hypogammaglobulinemia and mild prolonged activated partial thromboplastin time (APTT). Brain magnetic resonance imaging (MRI) revealed old lacunar infarction in the right basal ganglia and the lateral ventricle and fresh lacunar infarction in the right pons, respectively. The perivascular sheathing of FBA and macular edema were resolved after steroid administration and treatment of intravitreal anti-VEGF injection. During the period of follow-up, the patient subsequently suffered from recurrence of strokes, abnormality of coagulation function, sudden hearing loss of the left ear, and diplopia. His gene sequencing results demonstrated several deletion mutations in *ADA2*, and the diagnosis of DADA2 was eventually confirmed.

**Conclusions:** FBA represents a very rare ocular feature of DADA2 and may in some cases be the presenting manifestation. Therefore, ophthalmologists need to be aware of this rare autoinflammatory disease.

## Introduction

Frosted branch angiitis (FBA), a rare form of a vascular disorder, was first report on an immunocompetent 6-year-old Japanese boy in 1976 (1). Subsequent reports delineated the typical findings: acute visual loss, variable inflammation with perivascular sheathing, late staining of vessels usually without obstruction of blood flow, and a rapid response to systemic corticosteroids (2). In recent years, several unusual FBA cases associated with ophthalmic and systemic autoimmune/autoinflammatory disorders [e.g., Crohn's disease (3), Behcet's disease (4), and systemic lupus erythematosus (5)] were reported. Deficiency of adenosine deaminase 2 (DADA2) is a systemic autoinflammatory disorder caused by a loss-of-function (LOF) mutation in the cat eye syndrome critical region protein 1 (*CECR1*, also called *ADA2*) gene, of which the three major manifestations are vasculitis, immunologic manifestations, as well as hematologic features. Its major symptoms usually appear in early childhood (i.e., age <10 years) (6–8). Here, we present the first case of FBA-like vasculitis in an adult DADA2 patient who also carries a heterozygous mutation of blood coagulation factor XII (F XII).

## Case Report

**Figure 1:**
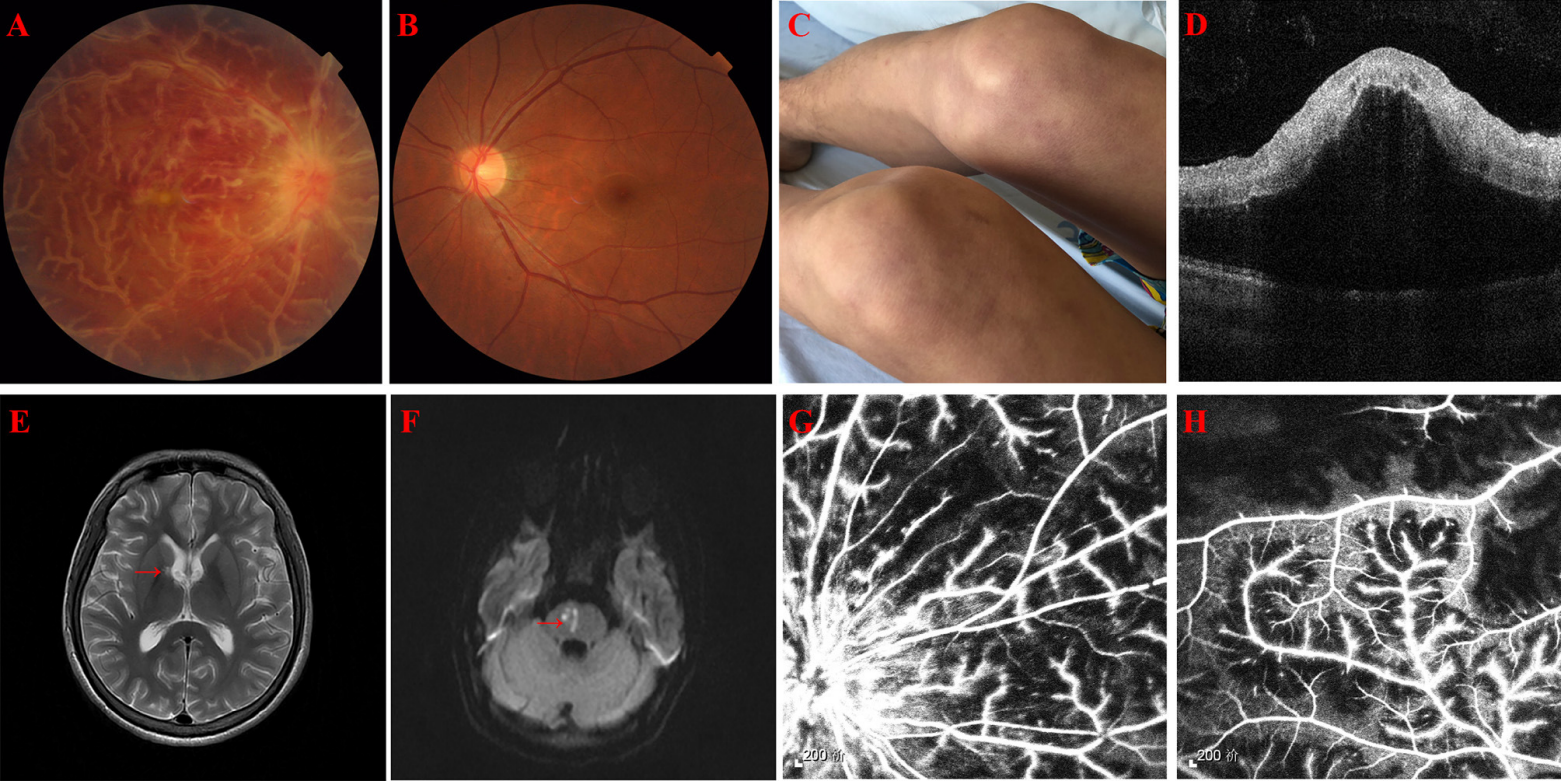
**(A)** A fundus photograph of the right eye showing thick, white confluent sheathing around retinal veins suggestive of frosted branch angiitis, disc hyperemia, and macular edema. **(B)** A fundus photograph of the left eye showing normal appearance. **(C)** Recurrent skin rash on both thighs. **(D)** Optical coherence tomography of the right eye showing exudative macular detachment, vitreous cells, and posterior vitreous detachment. **(E)** Old lacunar infarction involving the right basal ganglia and the lateral ventricle (red arrow) in magnetic resonance (MR) T2 phase, but not in the diffusion-weighted imaging (DWI) (image not showed). **(F)** Fresh lacunar infarction in the right pons (red arrow) in MR DWI. **(G,H)** Fluorescein angiography of the right eye showing extensive vascular leakage and supratemporal capillary non-perfusion areas.

A 31-year-old Chinese male with sudden onset of blurred vision in his right eye for 2 days was referred to our clinic on January 21, 2019. On examination, best corrected visual acuity (BCVA) was 20/20 and 20/1,000 in the left and right eye, respectively. Furthermore, intraocular pressure (IOP) was elevated to 39.3 mmHg in the right eye. A slit lamp demonstrated ciliary hyperemia, positive fresh keratic precipitates (KP), positive anterior chamber flare and cell, and positive relativistic afferent pupillary disorder (RAPD) in the right eye. On fundus examination, the right eye showed prominent white sheathing of retinal venules and, to a lesser extent, arterioles in all the quadrants, resembling the appearance characterized as “frosted branch angiitis.” Supratemporal retinal hemorrhages indicated concomitant branch retinal vein occlusion (BRVO). Moreover, macular edema and disc hyperemia were presented (Figure 1A). Fundus photograph of the left eye showed normal appearance (Figure 1B). Optical coherence tomography (OCT) suggested macular edema (Figure 1D). Fluorescein angiography (FA) revealed supratemporal bleeding-blocked fluorescence, concomitant capillary non-perfusion zone, and extensive dye leakage from vessels and the optic disc (Figures 1G,H). Positive anterior chamber flare and cells, together with retinal exudation and macular edema, also indicated panuveitis.

The patient has a history of intermittent and recurrent fever, skin rash (Figure 1C), and aphthous ulcer from 5 years ago. Tests from other hospitals implicated hypogammaglobulinemia with immune globulin (Ig) G 4.520 g/l, IgA 4.632 g/l, and IgM 0.134 g/l. One month ago, the patient visited a neurology clinic with complaints of dizziness, nausea, vomiting, totter, and slurred speech. Magnetic resonance imaging (MRI) revealed lacunar infarction in the right basal ganglia and the lateral ventricle (Figure 1E). Activated partial thromboplastin time (APTT) was mildly prolonged to 50.3 s. CBC showed elevated neutrophil percentage (NEU%) of 88.1% and decreased lymphocyte (LYM#) of 0.45 × 10^9^/L. Hepatorenal function, cranial angiography, carotid and cardiac ultrasound, and dynamic electrocardiogram were uneventful. Because of self-claimed allergy to aspirin, patient was administered clopidogrel and atorvastatin once a day.

With consultation from neurologists and rheumatologists, the following laboratory investigations were carried out: raised C reactive protein (CRP) of 41.2 mg/l, homocysteine of 16.0 μmol/l, decreased CD25 + lymphocyte of 4.06%, helper T-cell of 3.70%, IgG of 4.3 g/l, IgA of 0.48 g/l, and IgM of <0.25 g/l, and microcytic hypochromic anemia was noticed. Other workups, including urine routines, erythrocyte sedimentation rate (ESR), coagulation function, streptolysin O, rheumatoid factor, antiphospholipid antibodies, antineutrophilic cytoplasmic antibodies, antinuclear antibodies, protein C, protein S, *Toxoplasma gondii*, rubella, herpes simplex, hepatitis B, hepatitis C, Epstein–Barr virus, HIV, cytomegalovirus, *Treponema pallidum*, and *Mycobacterium tuberculosis*, were negative. Anterior chamber paracentesis for high-throughput sequencing did not find any pathogenic microorganisms.

**Figure 2:**
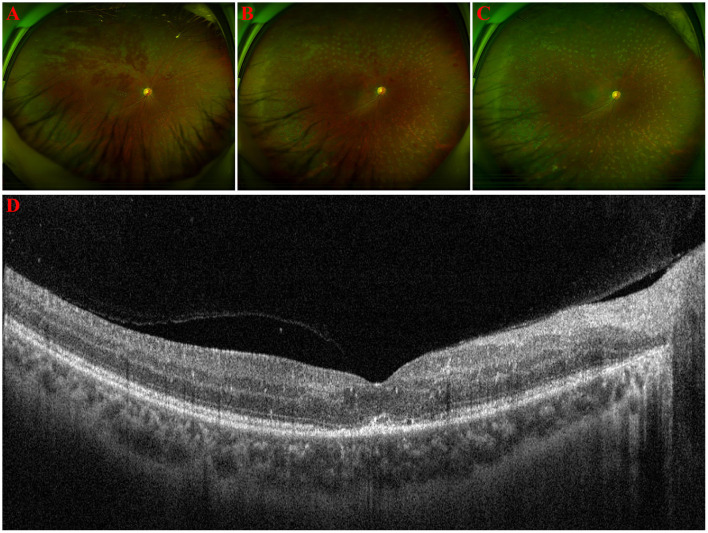
**(A–C)** Ultra-wide-angle fundus photographs of the right eye from 1, 2, and 5 months post-treatment. White sheathing of the vessels alleviated first, and supratemporal hemorrhage resolved at 5 months later. **(D)** Optical coherence tomography of the right eye 1 month after treatment showing disappearance of subretinal exudation and much less vitreous cells.

Oral methylprednisolone 24 mg/day was started and tapered off within 3 months, together with three times of periocular injection of 20 mg methylprednisolone. Topical fluorometholone (0.1%) and pranoprofen (0.1%) eye drops four times per day were administered. Carteolol (2%) and brinzolamide (1%) eye drops were used to alleviate IOP. Focal retinal photocoagulation and intravitreal injection of ranibizumab (0.5 mg/50 μl) were given to control fundus hemorrhage. One week after treatment, KP and aqueous flare and cells disappeared and the IOP decreased back to normal. One month later, the macular edema was resolved in the right eye (Figure 2D). White sheathing of the vessels alleviated (Figure 2A). Two months later, a fundus photo showed full resolution of vascular sheathing (Figure 2B) and visual acuity improved to 20/133. Retinal hemorrhage was absorbed at 5 months follow-up (Figure 2C).

**Figure 3:**
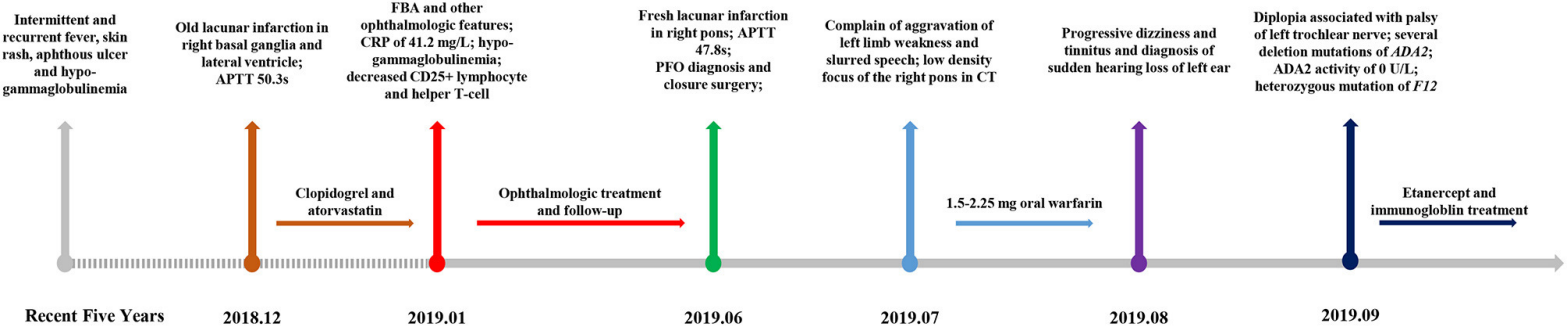
Time-line of main events of the patient.

However, the patient came to our emergency room (ER) with a complaint of left limb weakness and deterioration of slurred speech for 3 days, 5 months after being diagnosed of FBA. APTT was prolonged by 47.8 s, F XI and F XII activity was decreased to 66.7% (reference range: 70–120%) and 6.6% (reference range: 70–150%), respectively. MRI confirmed fresh lacunar infarction in the right pons (Figure 1F). The patient was diagnosed with patent foramen ovale (PFO) by contrast echocardiography of the right heart during admission and underwent a catheter-based closure surgery then. One month later, our patient was admitted in the emergency department of another center, complaining aggravation of left limb weakness and slurred speech. Due to the recent cardiac surgical implantation, cranial computerized tomography (CT) was adopted instead of MRI, which implicated low-density focus of the right pons. Considering deteriorating strokes under clopidogrel and atorvastatin intervention, 1.5–2.25 mg oral warfarin was given to prevent cryptogenic stroke, with international normalized ratio (INR) maintained between 2 and 3. One month later, the patient appeared with progressive dizziness and tinnitus and was diagnosed of sudden hearing loss of the left ear. Then, the patient presented with diplopia associated with palsy of the left trochlear nerve. Given the patient's complicated multiple disorders, whole exome sequencing (WES) was adopted to explore the possible pathogenesis. Several deletion mutations of *ADA2* (homozygous deletion of exon 7 and heterozygous deletion of exons 2–6 and 8–10) and heterozygous mutation of *F12* (p.K365Qfs^*^69) were recognized. Subsequent laboratory test demonstrated a total ADA activity of 2.2 U/l and an ADA2 activity of 0 U/l. Anti-TNF therapy was then started by weekly injection of 50 mg etanercept. Meanwhile, the patient received intravenous immunoglobulin according to monitoring. The brief timeline of our case is described in Figure 3.

## Discussion

FBA is a rare form of vasculitis characterized by florid translucent retinal perivascular sheathing, resembling the appearance of frosted tree branches in winter. Classical symptoms include sudden onset of blurred vision with floaters and photopsia. Despite the absence of a standard guideline treatment, most patients respond well with systemic steroids and have rapidly resolved with good visual recovery. Recurrence is rare. However, the prognosis has not always been favorable. Common complications include retinal vein/artery occlusion, fundus hemorrhage, optic disc atrophy, and macular edema (2).

The cause of FBA is unknown. Kleiner classified the patients with similar FBA appearance into three subgroups (9). First are the patients with lymphoma (10) or leukemia (11) whose disease is likely due to malignant cell infiltration. The second group consists of patients who have associated infections [including tuberculosis (12), syphilis (13), cytomegalovirus (14), HIV (15), rubella (16), Epstein–Barr virus (17), herpes simplex virus (18), etc.] or autoimmune diseases [such as Crohn's disease (3), Behcet's disease (4), systemic lupus erythematosus (5), etc.]. Inflammatory processes like vasculitis, immune complex deposition, or hypersensitivity reactions to microorganisms are considered to be the underlying mechanisms. The third group is composed of otherwise healthy young patients, who are probably undergoing immune response to underlying stimulus such as a viral infection. Nevertheless, until possible causative agents can be identified, the similar clinical appearances and courses were referred to as “acute idiopathic frosted branch angiitis.”

DADA2 is a systemic autoinflammatory disorder caused by LOF mutation in the *ADA2* gene, of which the three major manifestations are vasculitis, dysregulation of immune function, and hematologic disease (6–8). Inflammatory features include intermittent fevers, rash (often livedo racemosa/reticularis), and cutaneous ulcer. Vasculitis may manifest as early onset and recurrence of ischemic (lacunar) and/or hemorrhagic strokes or as cutaneous or systemic polyarteritis nodosa. Dysregulation of immune function can result in immunodeficiency or autoimmunity of varying severity. Hematologic disorders may begin early in life or rarely in late adulthood and can include lymphopenia, neutropenia, pure red cell aplasia, thrombocytopenia, or pancytopenia. Other clinical findings consist of musculoskeletal features, aphthous ulcers, inflammatory bowel disease-like illness, and hearing loss (19). To be noted, some manifestations of DADA2 might often overlap with polyarteritis nodosa (PAN), while others are more unique, especially immune deficiency and hematologic disease. Many clinical features or vessel histopathology do not appear to be helpful in differentiating DADA2 from PAN. Though there is a lack of guideline, gene and ADA2 functional evaluation is recommended in the diagnosis of DADA2 (19, 20).

Ophthalmologic manifestations have been noticed since DADA2 was first defined. Zhou et al. (21) reported ophthalmologic manifestations in five patients (total nine patients), including central retinal artery occlusion (CRAO) in one patient, optic nerve atrophy in one, diplopia with irregular enhancement of the medial rectus muscle (as indicated by cranial MRI) in one, third cranial nerve palsy in one, and strabismus in two. Furthermore, patients could have more than one ophthalmologic disorder. More recently, Sahin et al. (22) revealed ocular symptoms/signs in four patients (total eight patients), including temporary monocular/binocular vision loss in two patients, strabismus in two, panuveitis in one, and CRAO in one. These ophthalmologic features were likely to be contributed by possible central retinal artery involvement and third cranial nerve palsies after small initially undetectable strokes. Ocular inflammation (uveitis, scleritis, and episcleritis) (23), ptosis, and nystagmus were also documented (19, 24). So far, there have not been reports about a DADA2 patient displaying retinal vasculitis resembling FBA.

Experiments indicated that cecr1b is essential for both vascular integrity and neutrophil development in the zebrafish embryo and that both phenotypes are prevented by non-mutant, but not by mutant, human *CECR1* mRNA (21). DADA2 may compromise endothelial integrity while polarizing macrophage and monocyte subsets toward proinflammatory cells, establishing a vicious circle of vasculopathy and inflammation (25, 26), which is likely to trigger infiltration and exudation of retinal vessel presenting an FBA appearance. Our patient suffered from intermittent and recurrent fever, skin rash, lacunar infarctions, hypogammaglobulinemia, and aphthous ulcer and presented retinal vasculitis with hemorrhage, sudden hearing loss, and diplopia. A subsequent test confirmed deletion of several ADA2 exons with undetectable activity of ADA2 enzyme activity. Though no defined treatment strategies exist, anti-TNF agents (including etanercept, adalimumab, golimumab, infliximab, and certolizumab) have presented a remarkable effect on preventing ADA2-associated vasculitis (especially in reducing the risk of stroke) and, in some degree, relieving inflammatory burden. Hematopoietic stem cell transplantation (HSCT) can be curative to severe bone marrow abnormalities, which have poor response to anti-TNF agents. Steroids and general immunosuppressive therapies also have had variable success (27). In our case, the patient responded well to oral and topical administration of steroid without recurrence of perivascular sheathing and gained significant improvement of vision acuity. Elevated IOP and concomitant BRVO were also alleviated with the additional help of retinal photocoagulation, topical hypotensive, and anti-VEGF agents.

This patient also carried a heterozygous mutation of blood coagulation factor XII (F XII), which may lead to deficiency of F XII resulting in the development of thromboembolism rather than bleeding complications. A few investigators found that deficiency of F XII might be in association with elevated risk of retinal vessel occlusion (28, 29). However, other researchers hold a complete opposite opinion that mild F XII deficiency neither causes thrombosis nor protects from thrombosis (30–33). Based on current evidence and research basis, we could not ascertain whether the heterozygous mutation of F XII condition in our patient contributed to retinal vasculitis and other systemic symptoms or not. However, considering the heterozygous mutation of F XII and only mild prolonged APTT, it was not likely to play a vital role in this case.

To our best knowledge, this is the first case report of a patient with FBA-like retinal vasculitis due to adult DADA2. Although ADA2 is not expressed in endothelial cells, there is a defect in endothelial integrity in the small vessels of patients with ADA2 mutations as well as an impairment of M2 macrophage differentiation (34), which might cause the FBA appearance. In summary, FBA can be a presenting feature in DADA2 disorder, of which ophthalmologists need to be aware. Prompt treatment with corticosteroids and anti-inflammatory agents can lead to good remission.

## Data Availability Statement

The original contributions presented in the study are included in the article/supplementary material, further inquiries can be directed to the corresponding author/s.

## Ethics Statement

The studies involving human participants were reviewed and approved by The Second Affiliated Hospital of Zhejiang University School of Medicine Review Board and Ethics Committee (No. 2020-286). The patients/participants provided their written informed consent to participate in this study.

## Author Contributions

All authors listed have made a substantial, direct, and intellectual contribution to the work, and approved it for publication.

## Conflict of Interest

The authors declare that the research was conducted in the absence of any commercial or financial relationships that could be construed as a potential conflict of interest.
